# A Highly Functional Synthetic Phage Display Library Containing over 40 Billion Human Antibody Clones

**DOI:** 10.1371/journal.pone.0100000

**Published:** 2014-06-20

**Authors:** Marcel Weber, Emil Bujak, Alessia Putelli, Alessandra Villa, Mattia Matasci, Laura Gualandi, Teresa Hemmerle, Sarah Wulhfard, Dario Neri

**Affiliations:** 1 Department of Chemistry and Applied Biosciences, Institute of Pharmaceutical Sciences, ETH Zürich, Zürich, Switzerland; 2 Philochem AG, Otelfingen, Switzerland; Imperial College London, United Kingdom

## Abstract

Several synthetic antibody phage display libraries have been created and used for the isolation of human monoclonal antibodies. The performance of antibody libraries, which is usually measured in terms of their ability to yield high-affinity binding specificities against target proteins of interest, depends both on technical aspects (such as library size and quality of cloning) and on design features (which influence the percentage of functional clones in the library and their ability to be used for practical applications). Here, we describe the design, construction and characterization of a combinatorial phage display library, comprising over 40 billion human antibody clones in single-chain fragment variable (scFv) format. The library was designed with the aim to obtain highly stable antibody clones, which can be affinity-purified on protein A supports, even when used in scFv format. The library was found to be highly functional, as >90% of randomly selected clones expressed the corresponding antibody. When selected against more than 15 antigens from various sources, the library always yielded specific and potent binders, at a higher frequency compared to previous antibody libraries. To demonstrate library performance in practical biomedical research projects, we isolated the human antibody G5, which reacts both against human and murine forms of the alternatively spliced BCD segment of tenascin-C, an extracellular matrix component frequently over-expressed in cancer and in chronic inflammation. The new library represents a useful source of binding specificities, both for academic research and for the development of antibody-based therapeutics.

## Introduction

Monoclonal antibodies represent an important class of pharmaceutical biotechnology products and important research tools in chemistry and in life sciences [1], [2]. The advent of phage display library technology [3]–[5] allowed the facile isolation of fully human antibodies from large combinatorial repertoires. While libraries were initially created starting from antibody genes isolated from natural sources (e.g., B cells in peripheral blood, spleen and tonsils [6], [7]), there has been a growing interest in the construction of rationally designed synthetic antibody libraries, in which individual library members incorporate structural features which are beneficial for practical applications [8]. Such libraries may yield clones which are homogenous in terms of their biophysical properties and amino acid sequence (thus facilitating affinity maturation procedures [9]), with some additional desirable properties, such as protein A binding for affinity capture applications [10].

Over the last 15 years, we have described and extensively validated human antibody synthetic libraries, which featured antibodies in scFv format [11] capable of binding to protein A affinity supports [10]. These antibody libraries have been used as sources of useful binding specificities, including the monoclonal antibodies F8, L19 and F16, specific to the alternatively spliced EDA and EDB domain of fibronectin and the A1 domain of tenascin-C, respectively [9], [12], [13]. The three antibodies, which have been shown to selectively recognize stromal and neovascular structures in cancer [14], [15] and inflammation [16], are able to preferentially localize at sites of pathological angiogenesis *in vivo* and are currently being investigated in Phase I and Phase II clinical trials [17]–[19].

In particular we have described antibody libraries of increasing size over the years: ETH2 (3×10^8^ clones [13]); ETH2Gold (3×10^9^ clones [20]); PHILO-1 and PHILO-2 (3.1×10^9^ clones [21]), which were all based on the combinatorial randomization of judiciously selected amino acid residues in CDR3 loops of heavy and light chains, while keeping the rest of the antibody scaffold constant.

While the majority of the synthetic antibody libraries described so far are able to yield binders against the majority of the proteins chosen as targets, the ability to isolate various diverse antibodies in a relatively short period of time (1–2 weeks) remains an important research goal in this field, in order to increase our ability to generate binding specificities against different epitopes and with different functional properties [21].

Here, we describe the design and construction of a very large antibody phage display library (termed “PHILODiamond”), containing over 40 billion human antibodies. This is the largest antibody library ever produced in our lab and one of the largest synthetic antibody libraries described in the literature [22]. The new library was highly functional, as revealed by the observation that >90% randomly picked antibody clones can be expressed at acceptable levels. A side-by-side comparison of antibody selections, performed with the PHILODiamond library and with other libraries against more than 15 antigens, revealed that various binding specificities could be isolated against structurally diverse targets.

The PHILODiamond library differs from other synthetic libraries in terms of size and modular design, facilitating affinity maturation procedures [12], [23]. Furthermore, all antibody clones bind to protein A, thus facilitating purification and detection procedure [10]. We introduced a S52N mutation in the VH domain, since position 52 is the most frequently mutated solvent exposed residue in the CDR2 loop and since asparagine may favor both donor and acceptor hydrogen bonding interactions [21], [24], . Position 52 is often mutated into an asparagine residue in naturally occurring antibodies [24], [26].

In order to demonstrate library performance for biomedical research applications, we raised human monoclonal antibodies against the alternatively spliced BCD segment of tenascin-C, a highly conserved alternatively spliced protein fragment comprising the three fibronectin type-III homology domains B, C and D, which displays 86% sequence identity between mouse and man, respectively. The binding properties of clone G5, originating directly from library selections without affinity-maturation procedures, were characterized *in vitro* by Biacore analysis (revealing a dissociation constant K_D_ = 27 nM against the human antigen) and *ex vivo*, by immunofluorescence analysis of human and mouse tumor sections.

## Materials and Methods

### Library Construction and Cloning

**Figure 1:**
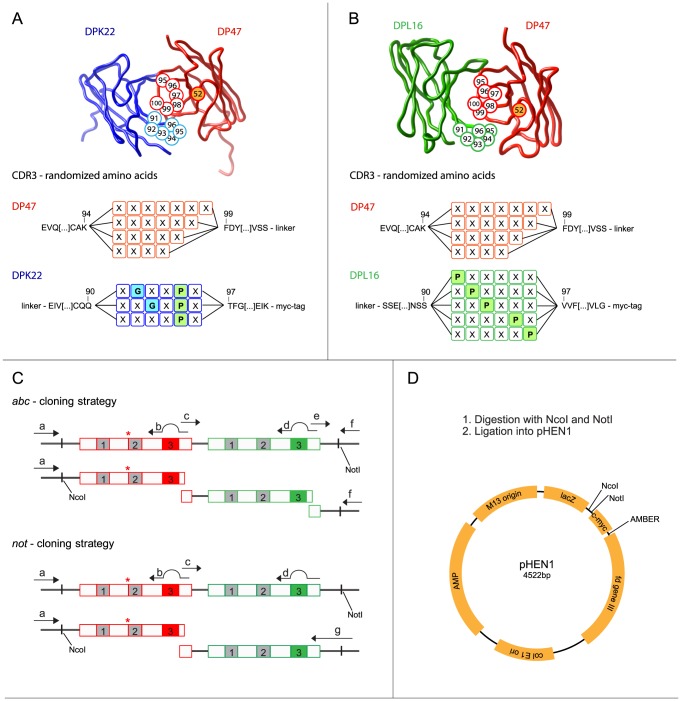
Design and cloning strategy of the PHILODiamond antibody library. (A, B) Three-dimensional structure of a scFv antibody fragment, with randomized amino acids highlighted in circles. In the DP47 heavy chain fragment (in red) position 95–100 were randomly mutated and the length of the CDR varies from 4 to 7 amino acids. A point mutation at position 52, short before the CDR2, was introduced converting a Ser to an Asn, as marked with a star. The antibody in (A) shows the DPK22 light chain fragment (in blue), in which the Pro95 was kept constant and a Gly residue was allowed to be located at position 92 or 93. The antibody in (B) contains a light chain based on the DPL16 germline segment. The CDR3 of light chain contains at least one Pro residue, either at position 91, 92, 93, 94 or 95. (C) Cloning strategy for the construction of different sub-libraries, which were eventually pooled to yield the PHILODiamond library. Primers are listed in Figure S2. Two different cloning strategies were used. In the *not-*strategy, two DNA fragments coding for the heavy or light chain respectively were assembled and amplified using a primer containing the *Not*I-restriction site. In the *abc-*strategy, an additional DNA segment, which contained the *Not*I-restriction site, was assembled together with the randomized light and heavy chain fragments. (D) All DNA fragments were amplified, double digested and ligated into the pHEN1 phagemid vector. All numbers in the antibody sequences are according to Tomlinson et al [43]. The three-dimensional structures were modelled from the Protein Data Bank files 1IGM (DPK22) and 8FAB (DPL16).

Two clones from PHILO-2 Library [21] with S52N mutation, one consisting out of DP47 and DPK22, the other one of DP47 and DPL16, were used as template for PCR amplification of the heavy chain DP47, and the light chains DPK22 and DPL16. Sequence variability in heavy and light chain was introduced in CDR3 loops by PCR using partially degenerated primers (**Figure S1**; all primers were purchased from Operon), as described earlier [20], [21] and shown in **Figure 1**. Briefly, DP47-based VH domains were randomly mutated from residue 95 to 100; this CDR3 loop was designed to be 4–7 amino acids long. DPK22-based VL domains were randomized between residues 91–96, with a fixed proline at position 95 and a glycine is either at position 92, at position 93 or neither at 92 or 93. DPL16-based VL domains were randomized between residues 91–96, with a proline at position 91, 92, 93, 95 or 96. Two strategies to assemble were used, named *not* and *abc*. Four sub-libraries were cloned using a primer including the *Not*I-restriction site on the primer. These were termed *not1k* and *not2k* and *not1l* and *not2l*, respectively for each light chain. For the *abc* sub-libraries, three parts, *(a)* for the heavy chain including a randomized CDR3, *(b)* for the light chain (DPK22 (k) or DPL16(l)) with the randomized CDR3 part and *(c)* a constant part including the *Not*I restriction site, were stoichiometrically assembled and amplified by PCR using strategy shown in **Figure 1** and primers listed in **Figure S1.** The resulting scFv genes were double digested with *Nco*I/*Not*I and ligated into the freshly *Nco*I*/Not*I-digested phagemid vector pHEN1 [27]. The ligation was purified and electroporated into freshly prepared electrocompetent TG-1 cells. Electrocompetent TG-1 cells were prepared by washing the cells, which are in exponential growing phase, twice with sterile 1 mM HEPES/5% glycerol and twice with sterile 10% glycerol in water. Finally, cells were resuspended in 10% glycerol to a density of approximately 10^11^ cells/ml. Electroporated cells were spread on 2xYT-agar plates with ampicillin and glucose (1 L: 16 g bacto-tryptone, 10 g bacto-yeast extract, 5 g NaCl, 15 g agar, 100 mg ampicillin, 1 g glucose) and incubated at 30°C overnight. On the next day, cells were rescued (with 2xYT−10% glycerol), used for phage production according to standard protocol [20] and stored as glycerol stocks. The different sub-libraries were electroporated on different days.

### Library Characterization

A total of 88 clones were tested by PCR screening with REDTaq Ready Mix PCR reaction mix (Sigma Aldrich) using primers [a] and [h] listed in **Figure S1.** Out of these clones, 55 were sequenced by Sanger sequencing (GATC Biotech) using primer [a] or [h]. For dot blot analysis, individual colonies from the plated library were inoculated in 160 µL 2xYT with ampicillin (100 µg/mL) and glucose (0.1%) in U-bottom 96-well plates (Nunc). The plates were incubated 4 h at 37°C in an orbital shaking incubator. Expression was induced by addition of 40 µL 2xYT containing IPTG to yield a final concentration of 1 mM and cultures were grown overnight at 30°C on an orbital shaker. ScFv-containing supernatants were blotted onto 0.45 µm nitrocellulose membrane (Santa Cruz Biotechnology) using the ELIFA system (Pierce). ScFv was detected with anti-myc tag murine antibody 9E10, followed by anti-mouse IgG horseradish peroxidase conjugate (Sigma Aldrich). Peroxidase activity was detected using the ECL plus western blotting detection system (Amersham Biosciences, GE Healthcare) on Amersham Hyperfilm ECL (Amersham Biosciences, GE Healthcare).

### Antibody Selection

Selections were performed according to standard protocols, using recombinant or purified antigen with high purity as assessed by SDS-PAGE and size exclusion chromatography. In brief, the antigen was either coated on MaxiSorp strips in 8×125 µL at around 5×10^−6^ M in PBS overnight, or the biotinylated antigen was coated on StreptaWells (Roche) with 8×125 µL with a concentration of around 10^−6^ M or on 60 µL streptavidin-coated beads M280 (life technologies) in a volume of 200 µL for 30 min at RT. The wells or beads were blocked using 2% w/v skimmed milk powder in PBS (MPBS). After rinsing with PBS, about >10^12^ phage particles were added to the antigen-coated surface in the presence of 2% MPBS, incubated for 2 h shaking (100 rpm) at RT. For the selections in wells after 30 min. the incubation was performed without agitation. Unbound phage were washed with PBS Tween 0.1% (7 to 20 times) and PBS (3 to 20 times), while bound phage were eluted with 100 mM triethylamine (TEA). Eluted phage were neutralized by adding 1M Tris HCl pH 7.8 and used for infection of exponentially growing *E. coli* TG1. After 2 rounds of panning, ELISA screening was performed with 94 individual colonies as previously described by Silacci and colleagues [20]. In brief, individual colonies were inoculated in 200 µL 2xYT, 100 µg/mL ampicillin (Fisher Bioreagents), 0.1% glucose (Sigma Aldrich) in Nunclon U-bottom 96-well plates (Nunc). The plates were incubated 3 h at 37°C in an orbital shaker incubator. The cells were then induced with isopropyl thiogalactopyranoside (IPTG; AppliChem) at a final concentration of 1 mM and grown overnight at 30°C. The bacterial supernatants containing soluble scFv were tested in ELISA experiments as described before [12] using the anti-myc tag 9E10 mAb and anti-mouse horseradish peroxidase (HRP) immunoglobulins (Sigma-Aldrich) as secondary antibodies. 60 µL BM-Blue POD substrate (Roche) were added to each well for detection by colorimetric reaction. The reaction was stopped by adding 30 µL of 1 M H_2_SO_4_. The absorbance was measured using a plate reader (VersaMax) by subtraction of value at wavelength 650 nm from 450 nm.

### Expression and Purification of ScFv

A single colony was used to inoculate 10 ml of 2xYT media containing ampicillin (100 µg/mL) and 1% glucose and were incubated at 37°C on an orbital shaker until they were dense (about an absorbance at OD_600 nm_ of 1). This preculture was diluted 1∶100 in 400 mL of 2xYT containing ampicillin and 0.1% glucose and grown at 37°C until the OD_600 nm_ reached 0.4. The cells were than induced by addition of IPTG to a final concentration of 1 mM and grown over night at 30°C. The scFv antibody fragments were purified over Protein A Agarose (Sino Biotechnology) and eluted with 100 mM triethylamine.

### Size-Exclusion Chromatography (SEC)

Purified antibody fragments were analyzed on ÄKTA FPLC (GE Healthcare) using a Superdex 75 10/300 GL or Superdex 200 10/300 GL column, for a scFv or small immune protein (SIP) antibody fragment, respectively. For further analysis, the monomeric fraction was collected.

### Surface Plasmon Resonance (SPR) Analysis

SPR was performed on a Biacore 3000 system (Biacore, GE Healthcare) using CM3, CM5 or SA chip (Biacore) coated with the desired protein at 10 µL/min flow rate. 20–30 µL of antibodies were injected at different concentrations. Regeneration of the chip was performed by injecting 5 µL of 10 mM HCl. For supernatant screening, the same chips were used, and 20 µL the filtered (0.22 µm) supernatant were injected.

### Expression of BCD Domain of TnC

Recombinant murine BCD domains were cloned into pQE12 vector, expressed in *E. coli* and purified over NiNTA resin as described earlier [28]–[30] for human BCD domain, the sequence was cloned into pUC119 vector, an additional AVI-Tag followed by the His-Tag was fused at the C-terminus of the protein, and the fusion protein was expressed in *E. coli.*

### Reformatting into SIP (Small Immune Protein) Format

The DNA of the scFv was assembled with the CH4-DNA of a human IgE and cloned into a pcDNA3.1 vector (Invitrogen) as described earlier [12], [23], [31]. Expression of the protein was performed as previously described [32] using transient gene expression (TGE). Six days after transfection, the SIP proteins were purified from the supernatant by affinity chromatography using Protein A Agarose (Sino Biological).

### Immunofluorescence on Tumor Section

Xenograft or murine tumors were excised, embedded in NEG-50 freezing medium (Thermo Scientific) and stored at −80°C until sectioning. An immunofluorescence analysis on tumor sections (10 µm) was performed. The slides were fixed for 10 min in ice-cold acetone, rehydrated with PBS and blocked for 1 h with fetal calf serum (FCS). The slides were incubated with SIP antibodies (250 nM in PBS - 1% BSA) for 1 h at RT. The staining of primary antibodies was detected using an anti-human IgE antibody produced in rabbit and in a next step an anti-rabbit Alexa Fluor 549 (each 10 µg/ml in PBS - 1% BSA) (Invitrogen) for 30 min at RT. Nuclei were stained with DAPI (Invitrogen) and vessels were stained using a rat anti-CD31 antibody and an anti-rat Alexa Fluor 488 conjugate (not shown in the pictures). Each step was followed by 3 washes with PBS. Slides were mounted with Fluorescent Mounting medium (Dako), and images were acquired with a Zeiss Axioskop 2 MOT Plus (Carl Zeiss AG). Image analysis was performed using AxioVision 4.7 image analysis software (Carl Zeiss AG).

### Ethics Statement about Animal Sections

All murine and xenografts used for this analysis were prepared on the basis of our Project License (42/2012) at ETH Zurich. The License was issued by the Veterinaeramt des Kanton Zuerichs, to the name of Dario Neri.

## Results

### Design and Cloning of the Antibody Library

To generate a large, stable and highly diverse library of functional antibody in the single chain variable fragment (scFv) format, we cloned scFv fragments with sequence diversity restricted to the CDR3 loops of both heavy and light chain into a phagemid vector. A cloning strategy based on PCR-assembly steps was adopted (**Figure 1**), which resulted in a total number of 4.1×10^10^ independent clones. We used human antibodies based on the scFv format, with the DP47 germline sequence for the heavy chain variable domain, which confers high thermal stability and protein A binding properties [10]. In full analogy to previous antibody libraries, we chose either DPK22 or DPL16 as germline genes for the light chain and we used the flexible polypeptide linker GGGGSGGGGSGGGG to connect VH and VL in the scFv antibody format [20], [21]. The DP47, DPK22 and DPL16 germline genes are frequently used in humans, representing 12, 25 and 16% of the antibody repertoire, respectively [33]. CDR3 loops in VH domains were allowed to contain 4, 5, 6 or 7 combinatorial mutated amino acids, while CDR3 loops in VL domains were randomized in 5 or 6 positions (**Figure 1**). Furthermore, residue 52 of VH domains was designed to be an asparagine, in order to facilitate hydrogen bonding interactions. The complete amino acid sequence of the designed antibody library can be found in **Figure S1**.

**Figure 2:**
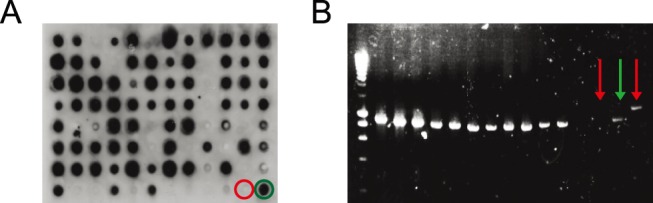
Quality controls on the antibody library. (A) Representative dot-blot analysis of 94 supernatants of individual unselected library clones. The soluble scFv fragments in the bacterial supernatants were detected using an anti-myc-tag antibody (9E10) and a secondary antibody coupled with HRP. As positive control, a purified scFv fragment with a myc-tag was loaded (green circle). As a negative control, 2xYT medium was used (red circle). (B) A representative agarose gel of the PCR colony screening of unselected clones from the library, used primers capable of amplifying the scFv insert. As a negative control, non-transfected bacteria (left red arrow) and bacteria containing pHEN1 vector with a longer insert (right red arrow) were used. As a positive control, a phagemid vector with a scFv insert was used (green arrow). All six dot-blots and PCR-screening gels related to the library construction process are shown in **Figure S2**.

The functionality of the library was initially verified by sequencing randomly picked clones, by studying the frequency of antibody-expressing clones and by PCR analysis of insert size. All unselected and sequenced clones (n = 55) showed a different CDR3 region in both heavy and the light chains. In dot-blot analysis, we found that more than 90% of the unselected clones were able to express scFv fragments, which could be detected with the anti-myc tag 9E10 antibody. PCR screening analysis revealed that 93% of the clones contained an insert of the right size (**Figure 2** and **Figure S2**).

### Antibody Selections

**Figure 3:**
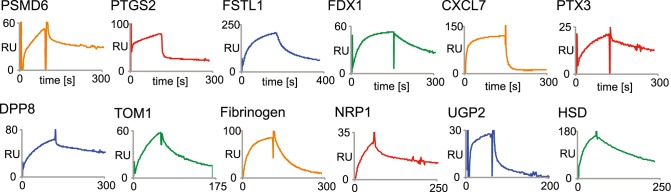
Biacore profiles of representative scFv fragments. The Biacore experiments were performed using monomeric preparations of scFv fragments specific to 12 different antigens (listed in the Figure) after bacterial expression, protein A purification and Superdex-75 gel filtration chromatography. The concentrations of the scFv were between 150 nM and 450 nM. RU: resonance units.

**Figure 4:**
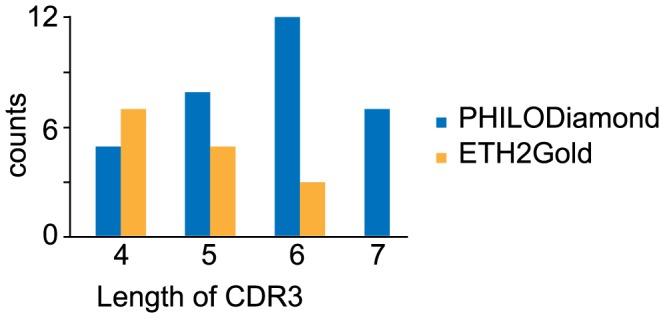
Analysis of the length of the randomized residues in CDR3 of the heavy chain from well characterized clones, isolated from the PHILODiamond and the ETH2Gold libraries against several antigens (selections performed in parallel). In the ETH2Gold library, the CDR3 loops of the heavy chain contained between 4 and 6 randomized consecutive amino acids, while in PHILODiamond between 4 and 7 amino acids were randomized. The PHILODiamond library exhibited a trend towards the isolation of scFv clones with longer CDR3 loops.

The PHILODiamond library was screened against a panel of more than 15 proteins, yielding positive clones against every target antigen, often with strongly positive clones already after two rounds of panning (**Table** **1**). Most of the binding antibody fragments were further analyzed by sequencing, gel-filtration and by surface plasmon resonance (SPR) analysis for K_D_ determination. Some SPR-profiles are shown in **Figure** **3**. K_D_ values typically ranged between 9 nM and 150 nM when measured with monomeric scFv fragment preparations, isolated after 2 or 3 rounds of panning. No obvious correlation could be observed between enrichment frequency, ELISA signal intensity and Biacore performance. Typically, scFv fragments may form non-covalent homodimeric structures (“diabodies”), contributing to functional binding affinity [34]. The distribution of lengths of randomized positions in VH CDR3 loops found in antibodies after antigen selection is displayed in **Figure 4**. The distribution reveals a preference for longer CDRs (six combinatorially mutated amino acids), compared to the results obtained with a synthetic library of similar design (ETH2Gold), previously reported by our group [20]. In CDR3 loops of VL domains, most binders had a preference for proline at position 95 or 96 of the randomized segment (**Figure S3**).

**Table 1:** Results of selections performed with the PHILODiamond library and with other libraries.

Antigen	PHILODiamond		ETH2 Gold		PHILO1–2	
	*ROP*	*Positive*	*ROP*	*Positive*	*ROP*	*Positive*
**Fibronectin (7B89)**	2	48	2	38	N.P.	N.P.
**Collagen I**	2	1	2	3	N.P.	N.P.
**Fibrinogen**	2	26	N.P.	N.P.	3	7
**Follistatin-like protein 1**	2	7	N.P.	N.P.	N.P.	N.P.
**Glutathione-S-transferase**	2	61	2	26	2	28
**Tenascin-C (BCD)**	2	30	2	35	N.P.	N.P.
**Human matrix metalloproteinase 1 (MMP1)**	2	16	N.P.	N.P.	N.P.	N.P.
**Human matrix metalloproteinase 3 (MMP3)**	2	18	2	23	N.P.	N.P.
**Mycolactone**	2	3	2	3	N.P.	N.P.
**Proteasome 26S (PSMD6)**	2	14	2	2	2	7
**Pentraxin-related protein 3**	2	14	N.P.	N.P.	N.P.	N.P.
**Serpin**	2	4	2	3	N.P.	N.P.
**Tissue inhibitor of metalloproteinase (TIMP)**	2	57	2	33	N.P.	N.P.
**Ubiquilin 1 (UBQL1)**	2	12	N.P.	N.P.	N.P.	N.P.
**Translocase of the outer membrane (TOM)**	3	3	2	3	N.P.	N.P.

In the left column, antigens that were used for selections are listed in alphabetical order. After 2 or 3 rounds of panning (ROP), 94 bacterial colonies were grown, induced in 200 µl 2xYT media and checked by ELISA for the presence of antigen-specific scFv fragments. The number of positive clones (out of the 94 colonies screened) corresponds to ELISA signals, which were higher than the signal of TG1 cell supernatant (negative control) by at least 20-fold. N.P.: not performed.

### Selection and Characterization of a Novel Monoclonal Antibody Specific to the Alternatively Spliced BCD Segment of Tenascin-C

In order to confirm that the PHILODiamond library was able to yield high-quality binders against proteins of pharmaceutical interest, we selected an antibody (termed G5) against the alternatively spliced BCD segment of tenascin-C. This fragment, which exhibits a 86% amino acid identity between mouse and man, is frequently over-expressed in cancer and in chronic inflammatory conditions [35], while being virtually undetectable in normal adult tissues [9].

**Figure 5:**
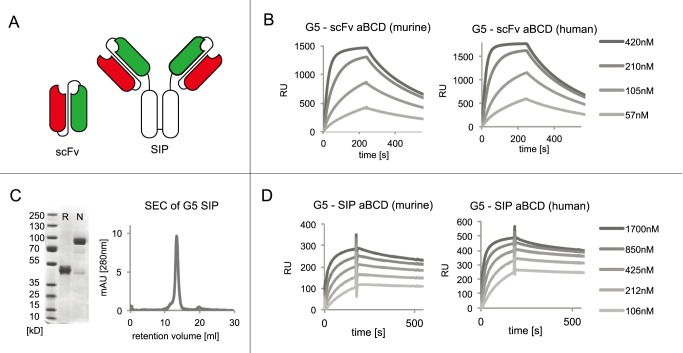
Characterization of the G5 antibody, specific to the alternatively spliced BCD domains of tenascin-C. (A) Schematic representation of the scFv and SIP antibody format. (B) Biacore characterization of the monomeric fraction of scFv (G5) produced at different concentrations on a CM5 Biacore microsensor chip, coated either with murine (left) or human recombinant BCD domains of tenascin-C (right). (C) SDS-PAGE of the G5 antibody in SIP format in reducing (R) and nonreducing (N) conditions, as well as gel-filtration profile on a S200 10/300 column. (D) Biacore profiles of the G5 antibody in SIP format, against murine and human recombinant BCD domains of tenascin-C.

The G5 antibody clone was isolated after two rounds of panning against a biotinylated version of recombinantly expressed murine BCD antigen [9] and reacted strongly with both murine and human cognate antigen. The complete amino acid sequence of the antibody clone is reported in **Figure S1**. The binding properties of the G5 clone in monomeric scFv format and in homodimeric SIP format [12], [36] against the human and the murine isoforms of BCD were analyzed by surface plasmon resonance on a Biacore instrument (**Figure 5**). The monomeric scFv antibody preparation exhibited similar kinetic binding constants towards the human and murine antigen (mouse: k_on_ = 7.9×10^4 ^s^−1^M^−1^; k_off_ = 2.5×10^−3 ^s^−1^; human: k_on_ = 1.1×10^5 ^s^−1^M^−1^; k_off_ = 3.0×10^−3 ^s^−1^), yielding a K_D_ value of 27 nM against human BCD and of 31 nM against murine BCD. SIP(G5), as expected, bound more avidly compared to monomeric scFv preparations, as a result of its homobivalent structure. The higher functional affinity of the SIP format is reflected in a slower dissociation from antigen coated on Biacore chip (**Figure 5**).

**Figure 6:**
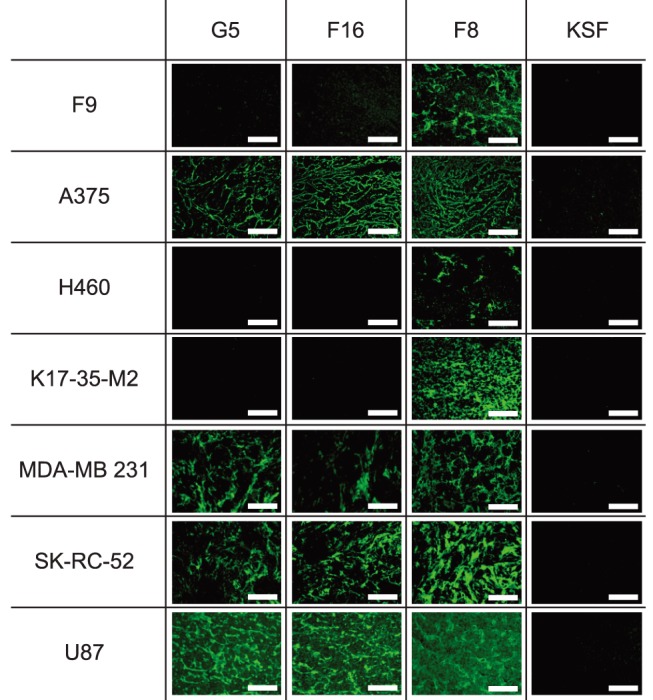
Immunofluorescence analysis of tumor sections. The staining properties of the G5 antibody were studied by immunofluorescence on frozen sections, derived from tumors grafted subcutaneously in mice. The staining results with G5 were compared to the ones of F16 (a clinical-state human antibody specific to the human, but not murine, alternatively spliced A1 domain of tenascin-C), F8 (a clinical-state human antibody specific to the alternatively spliced EDA domain of fibronectin, which recognizes the cognate antigen in mouse and man) and as a negative control KSF (specific to hen-egg lysozyme). All antibodies were tested in SIP format.

The ability of the G5 antibody to recognize the cognate antigen in normal and tumoral tissue, from both mouse and human origin, was assessed using immunofluorescence staining procedures on sections of freshly frozen specimens. G5 was compared to F8 and F16, two clinical-stage human antibodies specific to the alternatively spliced EDA domain of fibronectin and A1 domain of tenascin-C, respectively [9], [12]–[16], [19], [37], [38]. G5 revealed a staining pattern similar to the one of F16, with a strong reactivity against A375 (human malignant melanoma), MDA-MB 231 (human mammary gland), SK-RC-52 (human renal cell carcinoma) and U87 (human glioblastoma) tumors (**Figure 6**).

## Discussion

There is a growing interest in the use of antibody phage technology for the generation of fully human monoclonal antibodies. Phage display libraries differ in terms of antibody sequence, germline usage, randomization strategy, as well as library size and functionality. In this paper, we described a highly functional synthetic antibody displaying phage display library, containing over 40 billion clones. The library design incorporated germline sequences, which are often found in the human antibody repertoire and which were previously successfully incorporated in smaller but highly functional phage display libraries [20], [21]. The PHILODiamond library yielded specific binders against all protein antigens used as target (**Table 1**), including clones with K_D_ values <10 nM originating directly from library selections. As a representative, few SPR profiles are shown in **Figure 3**. In addition, the library yielded G5, an antibody fragment specific to the alternatively spliced BCD segment of tenascin-C, a marker of angiogenesis and of tumor stroma [29], which was studied in more detail, because of its possible biomedical applications. The antibody was found to bind human and mouse antigen with comparable affinity (**Figure 5**) and to strongly react with various types of tumors. By contrast, G5 did not stain virtually all normal adult tissues tested, exception made for eccrine sweat glands in skin and a weak staining in small intestine (**Figure S4**).

Synthetic naïve libraries are based on antibody genes, which are randomized at defined positions, while immunized libraries are based on VH and VL domains derived from an animal’s immune repertoire. Synthetic antibody libraries tend to yield clones with more homogenous properties and to perform better against highly conserved antigens, since antibody genes have not undergone *in vivo* negative selection [39]. We chose to concentrate amino acid-diversity in the CDR3 loops of heavy and light chains, since these positions are frequently involved in contact with the antigen [40]–[42]. However, we also chose asparagine as residue 52 of VH, as this position is frequently changed during somatic hypermutation [41],[43] and in affinity maturation procedures [44]. Library clones based on the DPL16 germline gene, we inserted at least one proline at positions 91, 92, 93, 95 or 96, according to previously published strategies [20], [21]. Sequence analysis of PHILODiamond-derived clones revealed a preference for a proline insertion at position 95 or 96, which may favor beta turn formation.

The DP47 germline VH gene, chosen for library construction, presents a number of attractive features. First, it is frequently associated with high thermal stability of the corresponding antibody clones [45]. Second, it confers binding to protein A even in scFv format, a feature, which is particularly attractive for antibody purification and for immunodetection purposes [10], [23], [46]. The PHILODiamond library was found to contain >90% of functional clones (**Figure 2** and **Figure S2**). It was tested on more than 15 antigens, ranging from big size molecules like collagen-I or fibrinogen, over a broad range of targets, including small catalytic domains (e.g., MMP3, TIMP or GST) and small toxic organic molecules (e.g, mycolactone) (**Table 1**). The modular design of the library allows a facile reformatting of antibody clones in several functional variants, such as SIPs or full length IgGs [36] Furthermore, the concentration of amino acid diversity in CDR3 loops facilitates the implementation of affinity maturation procedures by randomization of CDR1 or CDR2 region, as recently shown for the selection of antibodies against Placental Alkaline Phosphatase, an ovarian cancer marker [23].

We chose to use the scFv antibody rather than Fab fragments or dAbs, as scFv’s tend to express better and yield higher levels of antibody display on filamentous phage. On the other hand, scFv fragments may form non-covalent oligomers, a feature which is not shared by Fab fragments. Antibody clones based on scFv fragments can be easily reformatted into intact human immunoglobulins [31], [36], while the same feature is not possible with dAb-based antibodies, which lack the light chain domain.

The PHILODiamond library performed better for most antigens (e.g. GST, PSMD6 or TIMP), or at least in a similar fashion (e.g. for BCD, Serpin or Mycolactone), when compared in side-by-side selections with the ETH2-Gold library. Only in the case of collagen I, fewer antibody clones were isolated from the PHILODiamond library. The new library presents a number of attractive features, including binding to protein A for all library members. This property cannot be achieved using other synthetic libraries (e.g., [47]), which make use of various types of germline genes coding for the VH domain.

Tenascin-C is an extracellular matrix component, which exists in various splice isoforms. While the extra-domains A to D are absent in the small tenascin-C isoform, which is found in several healthy tissues, splice isoforms containing extra-domains exhibit a more restricted pattern of expression in normal organs. By contrast, large tenascin-C isoforms can be very abundant during embryogenesis, in cancer and in chronic inflammation [9], [16], [19], [29], [35], [48], [49]. The G5 antibody recognizes its cognate antigen in mouse and human specimens, with prominent stromal and vascular patterns of staining. As such, it is ideally suited for the development of antibody-based targeted biopharmaceuticals, which may carry cytotoxic drugs [50]–[53], radionuclides [54] or cytokines [55] as therapeutic payloads. Our group has recently reported promising examples of therapeutic activity in cancer patients for anti-tenascin-C antibodies, armed with interleukin-2 [19], [56] or with iodine-131 [57].

In summary, we have described a large and highly functional synthetic phage display library, which may be broadly useful for the isolation of antigen-binding specificities. The technology has been perfected over the years, to an extent that virtually any purified protein can be successfully used as target for antibody selections. The performance of the anti-tenascin G5 antibody was shown that affinity reagents, directly isolated from the library, perform well for biomedical research applications.

## Supporting Information

Figure S1 Used primers and sequence of SIP G5. (A) Primers used for the library construction. (B) Full length sequence of the anti-BCD antibody G5 in the SIP format.
(EPS)

Figure S2 Quality control of the six sub-libraries. (A) Dot blot analysis of 94 induced supernatants of individual unselected library clones. The soluble scFv were detected with an anti-myc-tag antibody (9E10) and a secondary antibody coupled with HRP. Positive controls are marked with a green circle. A red circle surrounds negative controls. The number below the library name represents the titer of individual clones in the sub-library. (B) Agarose gel of the PCR colony screening of unselected clones using primers annealing up- and down-stream of the scFv gene inserted into the pHEN1 vector. The number below the library names represents the number of clones carrying the right insert compared to total number of screened clones.
(EPS)

Figure S3 Analysis of the light chains of well-characterized antibodies with good affinity against different antigens. (A) According to the library design it is possible that a glycine is present at position 2 (1), at position 3 (5) or neither positions 2 or 3 (4). Some clones had more than a single glycine inside CDR3. At position 5 there is always a Pro. (B) The CDR3 of the DPL16 has a proline at position 1,2,3,5 or 6. Here the distribution is shown according to the position inside the CDR3.
(EPS)

Figure S4 Immunofluorescence of G5 anti-BCD antibody tested on a frozen tissue array from healthy humans (BioChain, T6234700-5, B403108). G5 antibody is shown in green, nuclei are stained with DAPI (shown in blue).
(EPS)
